# Unveiling the potential of marine peptides in leukemia: mechanistic insights and future horizon in peptide research

**DOI:** 10.55730/1300-0144.6061

**Published:** 2025-09-02

**Authors:** Salman AHMED, Waqas ALAM, Khalaf ALSHARIF, Michael ASCHNER, Esra KÜPELİ AKKOL, Mamdouh ALLAHYANI, Hayaa ALHUTHALI, Luciano SASO, Esra Gizem TÜRKCANOĞLU, Haroon KHAN

**Affiliations:** 1 Department of Pharmacognosy, Faculty of Pharmacy and Pharmaceutical Sciences, Karachi University, Karachi, Pakistan; 2 Department of Pharmacy, Abdul Wali Khan University Mardan, Mardan, Pakistan; 3 Department of Clinical Laboratory Sciences, College of Applied Medical Sciences, Taif University, Taif, Saudi Arabia; 4 Department of Molecular Pharmacology, Albert Einstein College of Medicine, New York, USA; 5 Department of Pharmacognosy, Faculty of Pharmacy, Gazi University, Ankara, Turkiye; 6 Department of Physiology and Pharmacology “Vittorio Erspamer”, Sapienza University, Rome, Italy

**Keywords:** Anticancer, marine peptides, mechanistic insights, MDR cancer, mitochondrial dysfunction, clinical status

## Abstract

Leukemia is a malignant disorder that affects the bone marrow and blood. It is frequently diagnosed in adults, with a higher incidence in males than females. Globally, it ranks as the 15th most widespread cancer and is the 11th leading cause of cancer-related fatalities. The intensity and severity of treatment have increased, leading to a rise in relapse rates, toxicity, and adverse health effects. Therefore, there is a critical need for new treatments. The antileukemic properties of natural compounds have been extensively studied. Aquatic organisms are a rich source of potential medicinal compounds. Anticancer peptides offer an ideal treatment approach due to their anticancer effects and reduced toxicity to normal cells. This review provides a comprehensive analysis of the diverse mechanisms through which marine-derived peptides exert their physiological effects, including modulation of cell viability, induction of apoptosis, cell cycle arrest, antimitotic and antimetastatic activities, immunostimulation, mitochondrial dysfunction, and oxidative stress. Additionally, these peptides target alterations in lipid composition and microtubule dynamics within cancer cell membranes. Notably, their efficacy has been proven in multidrug-resistant (MDR) leukemic cell lines, with evidence of synergistic effects when combined with conventional antileukemic therapies. Collectively, these findings support the potential of marine peptides as promising candidates for the development of novel antileukemic agents.

## 1. Introduction

Leukemia is a malignant disorder that impacts the blood and bone marrow. It is 10 times more frequent in adults than children and has a higher incidence in males than in females. Based on the latest data from the GLOBOCAN global cancer statistics for 2020, leukemia ranked as the 15th most diagnosed cancer globally. It was reported to account for 474,519 cases and 311,594 deaths, ranking as the 11th leading cause of mortality related to malignant diseases. Leukemia is a complex disease that has several subtypes and classifications. It can be categorized as either acute or chronic based on its pathological characteristics and rate of progression. In chronic leukemia, hematopoietic stem cells (HSCs) differentiate into partially functional cells, while in acute leukemia, HSCs remain immature and undifferentiated progenitor cells that produce blast cells. Different types of leukemia include acute and chronic lymphocytic leukemia (ALL and CLL, respectively), and acute and chronic myeloid leukemia (AML and CML, respectively) [1]. Conventional treatments, such as chemotherapy and stem cell transplantation, have improved survival rates for many leukemia patients. However, they can have significant adverse side effects, lead to chemoresistance, and may not be curative in all cases. As a result, researchers and clinicians are constantly exploring alternative approaches, including the use of natural products to prevent and treat leukemia. National Cancer Institute research on natural product extracts derived from plants and marine organisms has identified several candidates with promising therapeutic potential for leukemia treatment. Natural products contain complex mixtures of bioactive compounds with antiproliferative, proapoptotic (promoting cell death), antiinflammatory, antiangiogenic (inhibiting blood vessel formation), and immune-modulatory effects that are useful in treating leukemia. Furthermore, natural products can be combined with conventional chemotherapy drugs [2].

Marine organisms have shown great potential as a source of natural products with anticancer properties. Marine organisms inhabit physically ad chemically unique environments, leading to the synthesis of structurally diverse and biologically active compounds. Following its initial approval by the US Food and Drug Administration in 1969, additional marine-derived compounds have received approval as anticancer medications, including trabectedin and eribulin mesylate [3].

Protein fragments known as peptides offer unique health advantages, and marine-derived peptides have become more popular because of their potential as anticancer treatments. These peptides have several advantages over proteins and antibodies, including their small size, ease of production, ability to cross cell membranes, low potential for drug–drug interactions, minimal blood–brain barrier disruption, preferential targeting, and many chemical and biological properties. Furthermore, they have fewer adverse effects on the liver and kidneys due to their limited accumulation in these organs. However, anticancer peptides have poor pharmacokinetics, restricted bioavailability, short half-life, susceptibility to proteases, and undergo first-pass metabolism. Clinical research on cancer treatment is pertinent to peptides that promote apoptosis, obstruct angiogenesis and cell proliferation, have antioxidant effects, and destabilize microtubules [4, 5]. This review focuses on the mechanistic insights and overall clinical potential of various reported marine-derived peptides in the treatment of leukemia.

## 2. Marine peptides in leukemia

In recent years, there has been an increase in studies on marine natural materials for the treatment of leukemia [6]. Anticancer peptides with proven efficacy against leukemia have been isolated from cyanobacteria, bacteria, fungi, algae, ascidians, mollusks, sponges, polyps (*Hydra*), horseshoe crab, and frogs. A linear peptide is made up of a straight chain amino acids connected with amide bonds [7]. Linear tripeptides include criamide B, hemiasterlin B [8] and HTI-286 (taltobulin/SPA-110/hemiasterlin analog) [9]; linear tetrapeptides include discodermin E [10] and padanamides A and B [11]; linear pentapeptides include TZT-1027/soblidotin (dolastatin 10 derivative) [12]; a linear undecapeptide is koshikamide A2 [13]; and oligopeptides include proximicin B and C [14], simplicilliumtide A and G [15], polytheonamide A–C [16], yaku’amide A and B [17], arminin 1a–C [18], C-phycocyanin [19], magainin A, B, and G [20], malevamide D [21], polyphemusin I–III, and tachyplesin I–III [22]. Cyclic peptides have attracted the attention of researchers studying marine natural products as potential anticancer agents given their high binding ability, target specificity, minimal toxicities, ease of permeation of tumors, greater resistance to exo- and endopeptidases degradation, and higher bioavailability in vivo [23]. Cyclic tetratrideca-, thio- and depsipeptides have been reported to have antileukemic properties. Cyclic depsipeptides contain lactone bonds instead of amide groups that can be attributed to a hydroxylated carboxylic acid within the peptide structure [24]. Aurilide B–C [25], cryptophycin [26], cryptophycin-52 (LY355703) [27], hantupeptin A–C [28], kohamamide A–C [29], lagunamide A–C [30], largazole [31], trikoveramide A–C [32] from cyanobacteria; dolastatin 3, 10–15, and H [33, 34], kahalalide F [35], elisidepsin (irvalec/PM02734/kahalalide F derivative) [36], kulokekahilide-2 [37], and kulolide [38] from mollusk; didemnin A, B, and M, dehydro-didemnin B (aplidin/plitidepsin), nordidemnin, tamandarin B, and dehydrotamandarin A [39, 40] from ascidia; callipeltin A and B [41], geodiamolide A–F [42], halicylindramide A–C [43], homophymine A–E [44], jaspamide [45], and theopapuamide [46] from sponges; N-methylsansalvamide [47] and zygosporamide [48] from marine-derived fungi; and thiocoraline, BE-22179 [49], and romidepsin (FR 901228/FK 228) [50] from bacteria are the antileukemic cyclic depsipeptides. Asperterrestide A [51] and azumamide A (cyclic tetrapeptides) [52]; malformin A1 [53], motuporin [54], and nazumazole A–C (cyclic pentapeptides) [55]; cycloxazoline [56], kapakahine A–C [57], kapakahine E [58], and keenamide A (cyclic hexapeptides) [59]; axinastatin 2 and 3 [60], cupolamide A [61], mollamide [62], phakellistatins [63], stylopeptide [64], stylostatin 1 [65], trunkamide [66], and wainunuamide (cyclic heptapeptides) [67]; cycloforskamide [68], laxaphycin A and B1–B3 [69]; and piperazimycin A–C (cyclic hexadepsipeptide) [70] have been derived from ascidia, cyanobacteria, mollusks, sponges, and marine-derived bacteria and fungi. Thiopeptides are heterocyclic peptides with a 6-membered ring containing sulfur, known for their anticancer properties. Patellamide A–C and ulithiacyclamide [71] from ascidia and keramamide E–H and J–N from sponge [72] have antileukemic effects. Lipopeptides are peptides covalently linked with fatty acids [73]. Anabaenolysin A and B [74], dragonamide, pseudodysidenin [75], somocystinamide A [76], malyngamide S [77], lipodiscamide A–C [78], and sulfolipodiscamide A–C [79], are the lipopeptides from cyanobacteria, mollusk, and sponge with cytotoxic properties in leukemic cells. This review aims to highlight the efficacy and mechanisms of action of natural antileukemic marine peptides (Table 1).

## 3. Mechanistic insights

### 3.1. Apoptosis

Apoptosis is one approach to treating cancer. Loss of apoptosis regulation prolongs the life of cancer cells and promotes cell proliferation and angiogenesis. The release of cytochrome c (cyt c) from mitochondria into the cytoplasm in response to stress signals, such as DNA damage or oxidative stress, plays a vital role in apoptosis. When cyt c interacts with APAF1 in the cytoplasm, it forms a complex known as the apoptosome. Caspases are a series of proteolytic enzymes activated by the apoptosome and are primary mediators of apoptosis [80]. C-phycocyanin, derived from *Spirulina platensis* [19], and tachyplesin and cyclic tachyplesin are derived from *Tachypleus tridentatus* [81, 82]. All 3 compounds initiate cyt c release and promote apoptosis in K562 cells with IC50 values of 50, 1.7, and 2 μM, respectively. Caspases and poly ADP-ribose polymerase (PARP) are important in the apoptotic pathway, and their dysregulation can result in abnormal cell survival or death in leukemic cells. Following proteolytic cleavage, caspases are activated. Caspases-8, −9, and −10 trigger a regulated cascade of cell death, generating caspases-3, −6, and −7. PARP, an enzyme that contributes to genomic stability and DNA repair, is dysregulated in several types of cancer, such as leukemia. Leukemic cells can undergo apoptosis when caspases-3, −7, −8, and −9 are overexpressed or when PARP is inhibited [83]. Lagunamide A [30], C-phycocyanin [19], mere15 [84], tachyplesin, and cyclic tachyplesin [81, 82] increase caspase-3, −7, and −9 activity and PARP inhibition in HL-60 and K562 cells, thereby inducing apoptosis. Aplidin [39] and jaspamide [45] also promote caspase-3 activity and PARP inhibition in P388 and HL-60 cells. Bcl-2 inhibition and Bax activation have shown synergistic effects in preclinical studies, increasing apoptotic induction and boosting antileukemic activity [85]. Bcl-2 is a protein that inhibits apoptosis by preventing cyt c release. Bax, on the other hand, potentiates cyt c release and triggers apoptosis. In leukemia, cancer cells often overexpress Bcl-2, leading to apoptosis inhibition and increased cell survival [86]. Lagunamide A [30] and C-phycocyanin [19] from cyanobacteria, jaspamide from sponge [45], and mere15 from mollusks [84] increase Bax and decrease Bcl2 in HL-60 and K562 cell lines. Caspase-8-mediated apoptosis contributes to the antileukemic effect by promoting programmed cell death in leukemic cells. The downregulation of caspase-8 may contribute to tumor resistance to cytotoxic treatments secondary to reduced apoptosis [87]. Somocystinamide A [76] and tachyplesin [81, 82] induce apoptosis in CEM, Jurkat, MOLT-4, and K562 leukemia cells through the overexpression of caspase-8. Concurrently, inhibition of the PI3K/AKT signaling pathway decreases the phosphorylation of proapoptotic proteins Bad, Bax, and Bak, facilitating cyt c release, activation of caspase-9, and initiation of p53-dependent apoptotic pathways [88]. ErbB3 suppression causes cell cycle arrest and Bak and Bax activation [89]. Kahalalide F acts to reduce PI3K/AKT and ErbB3 as part of its anticancer action [35]. Topoisomerase inhibitors lead to DNA damage, inhibition of DNA repair processes, and activating caspases, ultimately resulting in apoptosis [90]. Patellin 6 (IC50 = 2 μg/mL) inhibit topoisomerase in P388 cells [91]. The myeloid cell leukemia 1 (MCL-1) protein is closely linked to carcinogenesis by apoptosis inhibition, a poor prognosis, and treatment resistance. Leukemia and other hematological cancers heavily rely on MCL-1 [92]. Lagunamide A and jaspamide inhibit MCL-1 in HL-60 leukemic cells [30, 45]. Optic atrophy 1 (OPA1) plays a critical role in maintaining mitochondrial integrity by preventing cyt c release and delaying apoptosis. Upregulation if OPA1 is also essential for angiogenesis and metastatic progression [93]. Prohibitin (PHB), an oncogenic protein, is associated with poor clinical outcomes and supports leukemic cell proliferation and differentiation [94]. Aurilide exerts its antileukemic effects by binding to and inhibiting PHB1, thereby promoting the proteolytic processing of OPA1 and inducing mitochondrial-mediated apoptosis in CCRF-CEM, HL-60, K562, and MOLT-4 cells [25]. Musashi-2 (MSI2), a stem cell-associated RNA-binding protein, is highly expressed in leukemia and is correlated with enhanced proliferation and reduced apoptosis; its downregulation leads to increased apoptosis and growth inhibition [95]. Tumor necrosis factor-related apoptosis-inducing ligand (TRAIL) selectively induces apoptosis in leukemic cells while sparing normal cells [96]. Largazole inhibits NB4 cell proliferation with an IC50 of 5 μM by significantly downregulating MSI2 expression and upregulating TRAIL levels [31]. Forkhead box M1 (FOXM1), a transcription factor essential for leukemic cell survival, promotes cell cycle progression; its inhibition leads to G2/M phase arrest [97]. Thiocoraline has been shown to downregulate FOXM1 in leukemic cells, contributing to its antiproliferative effects [98]. Aplidin induces apoptosis in K562 cells by targeting eukaryotic elongation factor 1-A2 (eEF1-A2) [99] and additionally inhibits ornithine decarboxylase (ODC), a key enzyme involved in polyamine biosynthesis and leukemic cell proliferation, in MOLT-4 cells [100].

### 3.2. Cell cycle arrest

**Figure 1:**
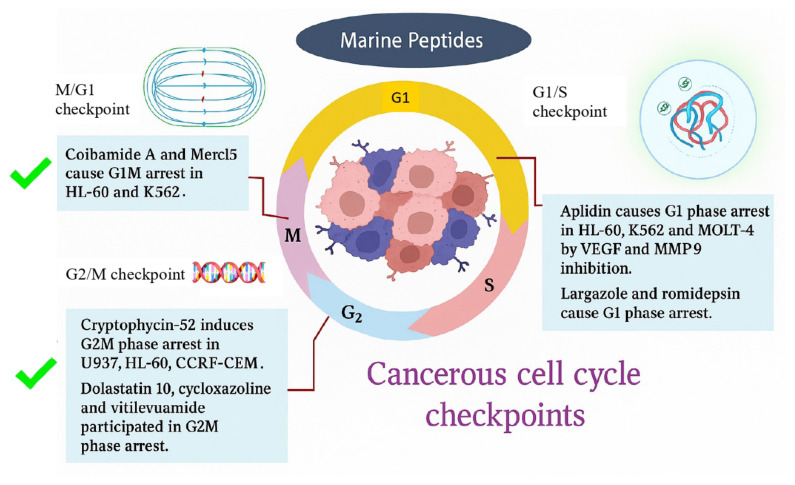
G1 and G2M phase arrest are crucial in maintaining genomic stability, impeding DNA-damaged cells from undergoing mitosis, maintaining proper cell cycle progression, mitigating aberrant cell division, facilitating apoptosis, and providing a target for cancer therapy in leukemic cell lines. Coibamide A and mere15 cause G1 and G0–G1 arrest in HL-60 and K562 cells. Cryptophycin-52 induces G2M phase arrest in U937, HL-60, CCRF-CEM. Similarly, dolastatin 10 and its derivative TZT-1027, cycloxazoline, and vitilevuamide induce G2M phase arrest against leukemic cell lines.

Cell cycle arrest is paramount in treating leukemia because it targets rapidly dividing leukemia cells, enhances chemotherapy efficacy, and enables targeted therapy. G1 and G2M phase arrest are crucial for maintaining genomic stability, hindering DNA-damaged cells from undergoing mitosis, maintaining proper cell cycle progression dodging aberrant cell division, and facilitating apoptosis, thus providing a target for cancer therapy in leukemic cell lines. Apoptosis eliminates DNA-damaged cells, thereby preventing the propagation of genetic mutations and reducing the risk of malignant transformation [101]. Coibamide A and mere15 [84] cause G1 and G0–G1 arrest in HL-60 and K562 cells, with IC50 values of 7.4 nM and 38.2 μg/mL, respectively. Cryptophycin-52 induces G2M phase arrest in U937, HL-60, and CCRF-CEM cells with IC50 values of 0.015, 0.018, and 0.022 nM, respectively [27]. Similarly, dolastatin 10 and its derivative TZT-1027 [12], cycloxazoline [56], and vitilevuamide [102] cause G2M phase arrest in leukemic cell lines. In leukemia, p21 overexpression causes G1 and G2M phase arrest and cell apoptosis [103] (Figure 1).

Marine peptides contribute to G1 and G2M arrest by p21 overexpression and cyclin D1 downregulation. Largazole and romidepsin are involved in G1 phase arrest [50]. Elevated levels of MMP-9 and VEGF may promote leukemic cell survival and growth. Aplidin causes G1 phase arrest in HL-60, K562, and MOLT-4 by VEGF and MMP-9 inhibition [40].

### 3.3. Histone hyperacetylation stimulation

The overexpression of histone deacetylases (HDACs) in leukemia makes them potential therapeutic targets to treat malignancy. HDAC inhibition (HDACi) initiates the DNA damage response, decreases cell proliferation (decreases cyclin D1, increases p21), induces apoptosis (increases caspases 3 and 9, decreases Bcl-xL), arrests the G2M phase, induces autophagy (increases mTOR, Beclin-1, and LC3), activates p53, and suppresses metastasis and angiogenesis (decreases VEGFR2 and MMP-2). Furthermore, HDACi also plays a vital role in overcoming multidrug resistance (MDR) [104]. Cyclic peptides largazole (NB4: 5 μM), azumamide A (K562: 4.5 μM) and romidepsin (P388: 0.37 nM) show cytotoxicity by HDACi [52].

### 3.4. Antimitotic/antimetastatic

Leukemic cells have the ability to disseminate via the bloodstream or lymphatic system to distant organs and tissues, where they may proliferate and form secondary tumors, a process referred to as metastatic leukemia.

Microtubule-targeting agents disrupt microtubule functions, making them promising candidates for antimetastatic therapy. Microtubules are critical for the formation of the mitotic spindle during cell division, which is essential for proper chromosome segregation and cytokinesis. Microtubule-targeting drugs inhibit mitosis and subsequent cell division by altering microtubule dynamics, often leading to cell cycle arrest or apoptosis. Additionally, these agents may impede angiogenesis, a process vital for tumor growth and metastasis. Due to the rapid proliferation of leukemia cells, they are particularly vulnerable to microtubule-targeting drugs, which disrupt microtubule dynamics and exert potent antimitotic effects. Microtubule-destabilizing agents induce apoptosis, G2M phase arrest, and metastatic incidents by upregulating proapoptotic Bax, Bad, and Bak and inactivating Bcl-2, Bcl-XL and MCL-1 [105]. Cryptophycin and its derivative cryptophycin-52 were cytotoxic in CCRF-CEM, HL-60, L1210, THP-1, and U937 cells by microtubule depolymerization [26, 27]. Dolastatin 3, 10–11, 13–15, H and dolastatin 10 derivative (TZT-1027) showed the same behavior in EHEB, JVM-2, HL-60, K562, L1210, and P388 cells in addition to inhibiting cancer cell growth in P388 mouse xenografts [12, 33, 34]. Mere15 from mollusk (*Meretrix meretrix*) caused microtubular depolymerization in K562 cells with IC50 of 38.2 μg/mL [84]. Vitilevuamide also contributed to the exact mechanism in P388 cells and P388 mouse xenograft [102]. Criamide B, hemiasterlin B, and hemiasterlin analog (HTI-286) are potent against P388 and CCRF-CEM cells via microtubule depolymerization [8, 9]. Actin microfilament polymerization promotes leukemic cell proliferation by preventing apoptosis, migration, and invasion of cancer cells in the presence of special AT-rich binding protein 1 (SATB1) [106]. Jaspamide cyclic depsipeptides from sponge (*Jaspis splendans*) possessed actin microfilament disruption in HL-60 (100 nM), Jurkat (2 μg/mL) and P388 (0.008 μg/mL) leukemic cells [45].

### 3.5. Mitochondrial dysfunction and oxidative damage

Oxidative stress is caused by reactive oxygen species (ROS) overproduction and impairment of endogenous protective antioxidant mechanisms. ROS exert deleterious effects that can compromise mitochondrial function. Mutations in mitochondrial DNA can impair the mitochondrial respiratory chain, leading to elevated production of ROS. In normal cells, multiple regulatory mechanisms maintain redox homeostasis by controlling intracellular ROS levels and protecting against oxidative damage to DNA, proteins, and lipids. In contrast, cancer cells typically have dysregulated redox balance, characterized by elevated ROS levels and compromised antioxidant defense systems that contribute to genomic instability and tumor progression. The elevated ROS levels and oxidative stress are critical in leukemia onset and progression [106]. Phycocyanin induces cytotoxicity in K562 by decreasing ROS [19]. DNA fragmentation is another evident consequence of oxidative stress, resulting in DNA damage and apoptosis. C-phycocyanin [19], jaspamide [45], TZT-1027 [12], romidepsin and proximicin B and C [14] cause DNA fragmentation in HL-60, Jurkat, K562, and P388 leukemic cell lines. Several mechanisms enable cancer cells to tolerate or repair DNA damage, ensuring sufficient DNA replication for continued proliferation. Thiocoraline, for instance, exerts anticancer effects by inhibiting DNA polymerase activity in P388 leukemia cells. Elevating reactive ROS levels has emerged as a potential anticancer strategy, as it induces oxidative stress, promotes DNA damage, disrupts cancer cell survival signaling pathways, and modulates immune responses. Cancer cells often have compromised antioxidant defenses, making them particularly susceptible to oxidative stress. Excessive ROS can overwhelm their redox capacity, leading to increased DNA damage, metabolic dysfunction, and ultimately cell death. Additionally, ROS can interfere with critical survival signaling cascades by inactivating proteins such as tyrosine phosphatases, thereby inhibiting proliferation and promoting apoptosis. ROS also play a role in enhancing antitumor immunity by stimulating T cell and natural killer (NK) cell activation, cytokine production, and cytotoxic responses. Many chemotherapeutic agents—including anthracyclines (e.g., doxorubicin, daunorubicin), alkylating agents, platinum-based drugs (e.g., cisplatin, carboplatin), and topoisomerase inhibitors (e.g., topotecan, irinotecan)—exert part of their efficacy by increasing ROS levels and disrupting redox homeostasis in malignant cells [107]. Marine peptides have been shown to exert antileukemic effects by increasing ROS production in leukemic cells, overwhelming their antioxidant defenses and causing oxidative stress. These effects can trigger a range of cellular responses, including DNA damage, inhibition of cell proliferation, induction of apoptosis (programmed cell death), and disruption of survival signaling pathways in leukemic cells. Lagunamides from cyanobacteria (*Lyngbya majuscula*) [30], mere15 from mollusk [84], aplidin from ascidia (*Aplidium albicans*) [39], and romidepsin from bacteria have been reported to have antileukemic effects through inducing ROS.

### 3.6. Immunostimulant

Immunostimulants are naturally occurring compounds that modulate the immune system by enhancing the immune response against cancer. Interleukin-2 (IL-2) is antileukemic and stimulates the immune system by activating NK and T cells—immune cells involved in the cytolysis of rapidly dividing abnormal cells. Tachyplesin and polyphemusin I–III promote immunostimulatory activity in HL-60 cells by mediating an intrinsic immune reaction via enhanced IL-2 [22].

### 3.7. Cancer cell membrane destruction by targeting its lipid composition

The development of novel therapeutic strategies targeting the lipid membranes of malignant cells aims to enhance their susceptibility to chemotherapeutic agents and overcome MDR. This approach is facilitated by the differences in lipid composition between the membranes of normal and tumor cells. Anticancer peptides exploit these differences to selectively disrupt tumor cell membranes, inducing necrosis and cell death. By depolarizing the cancer cell membrane, these peptides cause substantial cytoplasmic leakage, ultimately leading to cytotoxicity [108]. Arminin 1a-C was cytotoxic in HL-60, Jurkat, K562, and THP-1 by rapidly rupturing the cell membrane irreversibly, creating pores therein, and making it challenging to develop resistance [18]. Magainins A, B, and G irreversibly lyse leukemic cells K562, MLA, RPMI-8402, and SSKT1 with the exact mechanism, while normal cells remain unaffected [20]. Elisidepsin, tachyplesin, and polyphemusin showed similar effects in K562, MOLT-4, and HL-60 cells [22, 36]. Lipopeptides can induce apoptosis in tumor cells through multiple mechanisms, including pore formation leading to membrane leakage; modulation of membrane properties such as intrinsic curvature, elasticity, and fluidity; generation of ion channels; functioning as cation carriers; and exerting detergent-like effects on the lipid bilayer. These actions have the potential to induce cytotoxic effects [73]. Anabaenolysin A and B [74], somocystinamide A, dragonamide, pseudodysidenin [75, 76], malyngamide S [77], lipodiscamide A–C [78], and sulfolipodiscamide A–C [79] have been posited to contribute to cytotoxicity in CEM, HL-60, IPC-81, Jurkat, MOLT-4, NB4, and P388 leukemic cell lines.

### 3.8. Unidentified mechanisms for anticancer activity

Hantupeptin A–C [28], kohamamide A–C [29], laxaphycin A, B1–B3 [69], malevamide D [21], and trikoveramide A–C [32] from cyanobacteria; and keenamide A [59], kulolide [38], and kulokekahilide-2 [37] from mollusk have significant cytotoxic effects on several leukemia cells. However, the precise targets of these compounds are unknown. Cycloforskamide [68], lissoclinamide 4–6, patellamide A–D and F [71], mollamide [62], trunkamide [66], and ulithiacyclamide [69] from ascidia; and axinastatin 2 and 3 [60], callipeltin A and B [41], cupolamide A [61], discodermin E [10], geodiamolide A–F [42], halicylindramide A–C [43], homophymine A–E [44], kapakahine A–C [57], kapakahine E [58], keramamide E–M [72], koshikamide A and B [13], motuporin [54], nazumazole A–C [55], orbiculamide A [17], phakellistatin 1–16 [63], polytheonamide A–C [26], stylopeptide [101], theonellamide A–F [105, 106], theonellapeptolide [107], theopapuamide [81], and wainunuamide [103] from sponges also elicit antileukemic activity through an unidentified process.

Nyuzenamide A and B [109], padanamides A and B [11], and piperazimycin A–C [70], from marine-derived bacteria; asperterrestide A [51], malformin A1 [53], N-methylsansalvamide [47], simplicilliumtide A, E, G, and H [15], and zygosporamide [48] from marine-derived fungi elicit antileukemic properties through an unidentified mechanism.

## 4. Multidrug-resistant cancer

**Figure 2:**
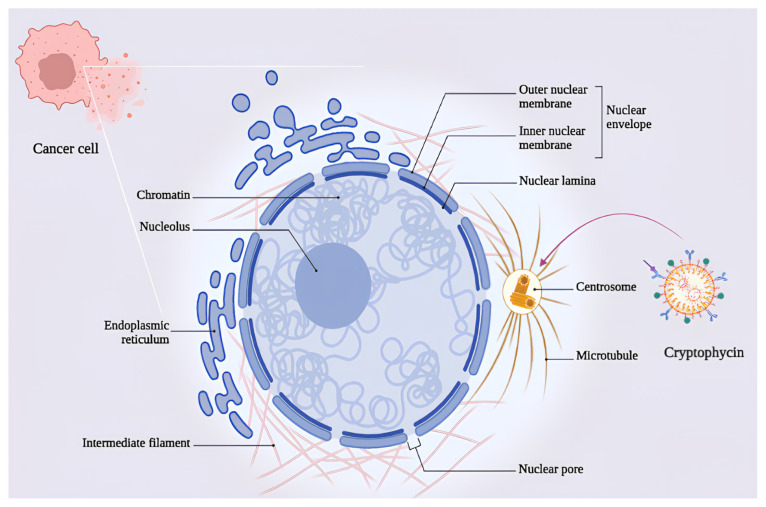
Cryptophycin is an antimicrotubule compound and a poorer substrate for P-gp in MDR cancers. Cryptophycin-52 (LY355703) is cytotoxic against P-gp overexpressed HL-60/VCR and multidrug resistance protein (MRP) overexpressed HL-60/ADR cell lines.

Drug resistance is one of the most frequent causes of treatment ineffectiveness in leukemic patients. P-gp, the most extensively researched efflux pump in MDR cancer, and other proteins including multidrug resistance protein (MRP), lipoprotein receptor-related protein (LRP), and breast cancer-related protein (BCRP) mediate the elimination of several anticancer drugs from leukemic cells [110]. TZT-1027 is cytotoxic against cisplatin, vincristine, and 5-fluorouracil-resistant p388 (P388/CDDP, P388/VCR, and P388/5FU). TZT-1027 causes microtubules depolymerization in P388/VCR (2 mg/kg, i.v.) and P388/5FU, P388/CDDP (1 mg/kg, i.v.) mouse xenografts. Cryptophycin is another antimicrotubular compound and a less favorable substrate for the P-gp in MDR cancers [111]. Cryptophycin-52 (LY355703) showed cytotoxicity against P-gp overexpressed HL-60/VCR and MRP overexpressed HL-60/ADR cell lines [45] (Figure 2).

Romidepsin showed cytotoxicity in P-gp over expressed P388/5FU and P388/MMC cells with IC50 values of 0.21 and 0.28 nM, respectively. The same cyclic depsipeptide (1 mg/kg, i.p.) inhibits MDR cell growth in P388/CPM, P388/MMC, P388/VCR and P388/5FU mouse xenografts [50]. Laxaphycin B is cytotoxic against MDR cancer cells CEM/VLB100 and CEM/VM-1 with IC50 values of 1.02 μM and 1.37 μM, respectively [69]. Homophymine A–E cyclic depsipeptides are effective against HL-60R cell lines [44]. Arminin 1a-C from polyps is efficient against K562/ADM with an IC50 of 14.10 μM [18]. FOXM1 overexpression has been implicated in chemotherapy resistance in leukemia. Thiocoraline has been suggested to promote apoptosis and enhance the efficacy of chemotherapy in leukemic cells through the suppression of FOXM1 [98].

## 5. Synergistic effects with other antileukemic agents

Marine peptides can enhance the antileukemic effects of other anticancer agents when given in combination. In the presence of patellamide D, the IC50 for vinblastine, colchicine, and adriamycin in CEM cells decrease from 100 to 1.5, 140 to 100 and >1000 to 110 ng/ml, respectively. Similarly, the IC50 for vinblastine against drug-resistant CEM/VBL100 cells decrease from 90 to 12 nM in the presence of patellamides B and C [112].

The combination of marine peptides laxaphycins A and B may hold therapeutic potential for leukemia. Laxaphycin A has a limited antiproliferative effect on leukemia cells. However, it significantly enhances the anticancer efficacy of laxaphycin B on both sensitive and resistant cancer cell lines. In the CCRF-CEM, CEM/VLB100, and CEM/VM-1 cell lines, the IC50 of laxaphycin B decrease as the concentration of laxaphycin A increases. In the presence of 0.2 μM of laxaphycin A, the IC50 value of laxaphycin B for CCRF-CEM cells decreases from 1.11 to 0.35 μM. Similarly, 0.2 μM of laxaphycin A decreases the IC50 values of laxaphycin B for MDR cancer cells CEM/VLB100 and CEM/VM-1 from 1.02 to 0.33 μM and 1.37 to 0.41 μM, respectively [69].

## 6. Clinical trial status

Aplidin is selectively cytotoxic against childhood leukemia cells in vitro. It does not show crossresistance with other cytotoxic agents, positioning it as a promising candidate for clinical trials. In 2003, the European Commission granted orphan drug designation for its use in treating acute lymphoblastic leukemia (ALL)^1^. The safety profile of aplidin has been thoroughly evaluated in phase I, II, and III clinical trials that reported transient, mild to moderate hematologic abnormalities that were well tolerated. In December 2018, aplidin received approval in Australia for the treatment of leukemia, multiple myeloma, and lymphoma, and was subsequently marketed by PharmaMar [114].

Dolastatin 10 is potent and effective in preclinical models, and has been through phase I and II antileukemic clinical investigations. Nonetheless, Phase III trials were halted given its toxicity. The dolastatin derivatives TZT-1027 (auristatin PE and soblidotin), cemadotin (LU103793), and tasidotin (ILX-651) have superior activity compared to the parent compound. Further research is required to evaluate their efficacy in clinical trials for the treatment of leukemia [115]. Another dolastatin derivative AGS67E (monomethyl auristatin E, MMAE) may play a role in leukemia treatment as an antibody–drug conjugate (ADC). AGS67E is conjugated with a distinctive monoclonal antibody to form brentuximab vedotin and zilovertamab vedotin [116].

Combined treatment regimens have been implemented to mitigate the toxicity of romidepsin consumption (e.g., HDACi). In a phase I trial in individuals with relapsed/refractory CLL, the maximum tolerated doses (MTDs) for bortezomib and romidepsin were 1.3 mg/m^2^ and 10 mg/m^2^, respectively, and dose-limiting toxicities were grade 3 tiredness, vomiting, and chills. The treatment regimen had minimal activity. The combination of romidepsin and azacitidine therapy trial in adults with relapsed AML successfully determined the MTD for safe and clinically effective treatment with romidepsin (12 mg/m^2^) and azacitidine (75 mg/m^2^) [117] (Table 2).

## 7. Conclusions and future perspectives

Leukemia is the 15th most common cause of cancer incidence and 11th most common cause for cancer-related death. Despite the efficacy of current antileukemic medications, their side effects, frequent relapses, and MDR mean that novel therapies are urgently needed.

Despite the global impact of leukemia, there is a notable lack of information in the literature regarding the use of marine peptides as a therapeutic strategy against this disease [2]. The antileukemic activities of marine peptides encompass cell growth inhibition, apoptosis induction, cell cycle arrest, stimulation of histone hyperacetylation, antimitotic, and antimetastatic effects as well as immunostimulation and disruption of cancer cell membranes. These peptides present a compelling and promising avenue for the development of antileukemic drugs and for identifying new therapeutic cellular targets. However, despite the potential, insufficient progress has been made, and further investigation into the antileukemic properties of marine peptides is necessary to identify novel molecules with therapeutic potential. Consequently, the antileukemic effects of various marine cytotoxic or anticancer peptides warrant further exploration.

Moreover, due to the scarcity of data on this topic, evaluating the toxicity and adverse effects of marine peptides on normal cells is crucial [118]. Assessing the efficacy of marine peptides is challenging, as most studies have been conducted in vitro, with only a few clinical investigations. The lack of both in vivo and clinical studies, combined with an incomplete understanding of their mechanisms of action, highlights the need for future research in this field. Even though some marine peptides with pharmacological activity have been excluded from drug discovery due to their toxicity, there remains substantial potential for the synthesis of suitable analogs to discover innovative pharmaceuticals from this rich source. The limited progress achieved so far has nevertheless yielded significant findings, particularly in structure-activity relationship studies.

Addressing formulation and administration challenges early on is critical when exploring novel peptides as potential anticancer therapies. This proactive approach can prevent setbacks in drug development related to unfavorable pharmacokinetics or toxicity outcomes and improve the likelihood of attracting attention from the pharmaceutical industry. Additionally, investigating the effects of marine peptides in combination with traditional chemotherapy, targeted therapy, and immunotherapy is essential. Novel techniques should also be used to isolate and identify anticancer marine peptides [4, 5].

Although marine peptides show considerable promise as antileukemic agents, several limitations must be addressed, including restricted availability, lack of standardization, safety concerns, incomplete understanding of mechanisms, regulatory challenges, potential for drug resistance, and issues related to cost and scalability. Therapeutic peptides face common drawbacks such as a short half-life, poor bioavailability, manufacturing difficulties, and susceptibility to proteases. To overcome these challenges, cell-penetrating peptides can be used to enhance cell membrane permeability. Approaches like D-amino acid substitution, cyclization, nanoparticle encapsulation, PEGylation, and XTEN conjugation can address metabolic instability and the short half-life of peptides. Moreover, substituting L-amino acids with D-amino acids may reduce immunogenicity [119].

Marine peptides show considerable promise in leukemia research due to their antileukemic effects, unique chemical structures, abundant marine biodiversity, low toxicity, and ability to synergize with other drugs. Further research in this area could lead to the discovery of new marine peptides with potent antileukemic properties, providing the foundation for innovative treatments for leukemia. In-depth studies of the mechanisms of action of various marine peptides would offer valuable insights for developing potential drug candidates to combat this deadly cancer.

## Figures and Tables

**Table 1:** Antileukemic effects of marine peptides.

Mechanism of action	Peptides (Marine source )
Apoptosis	Aplidin (*Aplidium albicans*) [39], Aurilide (*Lyngbya majuscula)* [25], C-phycocyanin (*Spirulina platensis*) [19], Jaspamide *(Jaspis johnstoni)* [45], Kahalalide F *(Elysia rufescens)* [35], Lagunamide A (*Lyngbya majuscula)* [30], Lagunamide A (*Lyngbya majuscula)* [30], MERE15 (*Meretrix meretrix*) [84], Patellin 6 (*Lissoclinum patella*) [91], Somocystinamide A (*Lyngbya majuscula*) [76], Tachyplesin (*Tachypleus tridentatus*) [81, 82]
Cell cycle arrest	Coibamide A [84], Cryptophycin-52 (*Nostoc* sp.) [27], Cycloxazoline (*Lissoclinum bistratum*) [56], Dolastatin 10 (*Dolabella auricularia)* [12], Largazole *(Symploca* sp.) [50], MERE15 (*Meretrix meretrix*) [84], Romidepsin (*Chromobacterium violaceum* No. 968.) [50], TZT-1027 (*Dolabella auricularia)* [12], Vitilevuamide *(Didemnum cuculiferum* and *Polysyncranton lithostrotum*) [102], Azumamide A (*Mycale izuensis*) [52]
HDAC inhibition	Largazole *(Symploca* sp.) and Romidepsin (*Chromobacterium violaceum* No. 968.) [52]
**Antimitotic/Antimetastatic**	Criamide B (*Cymbastela* sp.) [8, 9], Cryptophycin-52 (*Nostoc* sp.) [26, 27], Hemiasterlin B (*Cymbastela* sp.) [8, 9], HTI-286 (*Hemiasterella minor*, *Auletta* sp., *Cymbastela* sp., *Siphonochalina* sp.) [8, 9], Jaspamide *(Jaspis johnstoni)* [45], Mere15 (*Meretrix meretrix*) [84], TZT-1027 (*Dolabella auricularia)* [12, 33, 34], Vitilevuamide *(Didemnum cuculiferum* and *Polysyncranton lithostrotum*) [102]
**Mitochondrial dysfunction and oxidative damage**	Aplidin (*Aplidium albicans*) [39], C-phycocyanin (*Spirulina platensis*) [19], Jaspamide *(Jaspis johnstoni)* [45], Lagunamides (*Lyngbya majuscula*) [30], Mere15 (*Meretrix meretrix*) [84], Romidepsin (*Chromobacterium violaceum* No. 968.) and Proximicin B-C *(Verrucosispora* sp.) [14], TZT-1027 (*Dolabella auricularia)* [12]
**Immunostimulant**	Polyphemusin I–III and Tachyplesin *(Tachypleus tridentatus)* [22]
**Cancer cell membrane destruction by targeting its lipid composition**	Anabaenolysin A–B *(Anabaena* sp.) [74], Arminin 1a-C (*Hydra*) [18], Dragonamide (*Lyngbya majuscula*) [75, 76], Elisidepsin *(Elysia rufescens)* [22, 36], Lipodiscamide A–C (*Discodermia kiiensis*) [78], Magainins A, B and G (*Xenopus laevis*) [20], Malyngamide S (*Bursatella leachii)* [77], Polyphemusin *(Tachypleus tridentatus)* [22, 36], Pseudodysidenin and Somocystinamide A (*Lyngbya majuscula*) [75, 76], Sulfolipodiscamide A–C (*Discodermia kiiensis*) [79], Tachyplesin *(Tachypleus tridentatus)* [22, 36]

**Table 2:** Clinical trial status information on antileukemic marine peptides (from ClinicalTrials.gov).

Peptides	Clinical Trials Gov Identifier	Clinical Trial	Status	Study Completion Date
AGS67E (Monomethyl auristatin E, MMAE)	NCT02610062	Phase 1	Terminated	November, 2017
Aplidin	NCT00884286	Phase 2	Completed	June, 2010
Aplidin and Cytarabine	NCT00780143	Phase 1	Terminated	June, 2009
		Phase 2		
Brentuximab vedotin (SGN-35, monomethyl auristatin E)	NCT01461538	Phase 2	Completed	December, 2014
Dolastatin 10	NCT00003693	Phase 1	Completed	October, 2001
	NCT00005579	Phase 2		February, 2003
Romidepsin	NCT00053963	Phase 1	Completed	February, 2006
	NCT00024180			August, 2006
	NCT00042822	Phase 2		March, 2005
	NCT00062075			March, 2007
Romidepsin and Bortezomib	NCT00963274	Phase 1	Completed	April, 2018
Romidepsin and Decitabine	NCT00114257	Phase 1	Completed	September, 2006
*Zilovertamab vedotin* (VLS-101, MK*-*2140, Monomethyl auristatin E)	NCT03833180	Phase 1	Active or not recruiting	January, 2023 (study posted)

## Footnotes

1 European Medicines Agency Public Summary of Opinion on Orphan Designation: Aplidine for the Treatment of Acute Lymphoblastic Leukaemia [online]. Website https://www.ema.europa.eu/en/documents/orphan-designation/eu/3/03/151-public-summary-positive-opinion-orphan-designation-aplidine-treatment-acute-lymphoblastic_en.pdf [cited 2023 April, 29].
